# Phonological performance measured by speech severity indices compared with correlated factors

**DOI:** 10.1590/S1516-31802007000600002

**Published:** 2007-11-01

**Authors:** Haydée Fiszbein Wertzner, Luciana Amaro, Daniela Evaristo dos Santos Galea

**Affiliations:** Language-Speech-Hearing Sciences Course, Department of Physiotherapy, Language-Speech-Hearing Sciences and Occupational Therapy, Faculdade de Medicina da Universidade de São Paulo (FMUSP)

**Keywords:** Diagnosis, Language development disorders, Respiratory tract infections, Otitis, Severity of illness index, Diagnóstico, Transtornos do desenvolvimento da linguagem, Infecções respiratórias, Otite, Índice de gravidade de doença

## Abstract

**CONTEXT AND OBJECTIVE:**

Some factors seem to influence speech impairment among phonologically disordered children. The aim was to compare severity indices with some correlated factors.

**DESIGN AND SETTING:**

Observational, analytical and cross-sectional study conducted within the Language-Speech-Hearing Sciences Course, Universidade de São Paulo.

**METHOD:**

Fifty phonologically disordered children with ages ranging from 4 to 11 years took part. The indices were calculated from phonology tests and were correlated with anamnesis and audiological data. Student's t test and Spearman's correlation were used to compare percentages of consonants correct (PCC) and process density index (PDI) for children with and without otitis, upper respiratory histories and audiological abnormalities, with regard to whether or not they were comprehended during assessment, their ages when they started to speak and their ages at the assessment.

**RESULTS:**

The higher the age at the assessment was, the higher the PCC (imitation: 0.468; naming: 0.431; Spearman's correlation) and the lower the PDI (imitation: 0.459; naming: 0.431); the later the child started to speak, the lower the PCC (imitation p = 0.064; naming p = 0.050) and the higher the PDI (imitation p = 0.067; naming p = 0.042). There were differences between groups with and without upper respiratory history (PCC: imitation p = 0.016, naming p = 0.005; PDI: imitation p = 0.014, naming p = 0.008). There was no difference between the groups regarding otitis, comprehension during the assessment and audiological data.

**CONCLUSIONS:**

Children with upper respiratory histories who began to speak later presented more severe speech impairment indices.

## RESUMEN

**CONTEXTO E OBJETIVO:**

Alguns fatores parecem influenciar a gravidade dos distúrbios de fala em crianças com transtorno fonológico. O objetivo foi comparar índices de gravidade e fatores correlatos.

**DESENHO E LOCAL:**

Observacional, analítico, transversal. Curso de Fonoaudiologia, Universidade de São Paulo.

**MÉTODO:**

Cinqüenta crianças com transtorno fonológico, entre 4 e 11 anos. Calcularam-se os índices nos testes de fonologia e correlacionaram-nos com dados de anamnese e audiometria. O teste t de Student e a correlação de Spearman compararam a Porcentagem de Consoantes Corretas (PCC) e a Densidade de Processos Fonológicos (DPF) em crianças com e sem otite e história de infecções de vias aéreas superiores, alteração de audiometria, se eram ou não compreendidas na época da avaliação e quando começaram a falar e a idade em que começaram a falar e na avaliação.

**RESULTADOS:**

Quanto mais velha a criança na avaliação, maior a PCC (correlação de Spearman: 0,468, imitação; 0,431, nomeação) e menor a DPF (0,459, imitação; 0,431, nomeação); quanto mais tarde a criança começou a falar, menor a PCC (imitação p = 0,064; nomeação p = 0,050) e maior a DPF (imitação p = 0,067; nomeação p = 0,042). Observaram-se diferenças nas crianças com e sem infecções de vias aéreas (PCC: imitação: p = 0,016; nomeação: p = 0,005; DPF: imitação: p = 0,014 e nomeação: p = 0,008). Não houve diferença quanto à otite, compreensão na avaliação e audiometria.

**CONCLUSÃO:**

Crianças com história de infecções de vias aéreas e que começaram a falar mais tarde apresentaram maior comprometimento de índices de gravidade.

## INTRODUCTION

Despite the great occurrence of phonological disorders among children, their cause has not yet been defined. These disorders are characterized by abnormal sound production and inadequate use of the phonological rules of the language regarding sound distribution and syllable types. Furthermore, they present varying degrees of severity and speech intelligibility.^1^ Several studies have tried to correlate the linguistic symptoms presented by such subjects with possible variables that would interfere with the acquisition and mastery of phonological rules, and which could be the cause of these disorders.

The relationship between otitis and phonological disorders has also been studied. This is of interest because otitis is common in early infancy and decreases with increasing age. Otitis may occur in repeated episodes in many of these children.^2^ Researchers who have claimed that otitis has no effect on language have argued that, during otitis episodes, the conductive hearing loss ranges from mild to moderate and fluctuates, and that, immediately after the episode, the hearing returns to normal and the effects of the otitis are compensated by normal hearing.

Speech and language impairment is associated with the presence of otitis media with effusion, particularly when it appears during the first two years of life. Some authors have also expressed concern that other upper respiratory disorders, such as rhinitis, may interfere in the acquisition of language and speech.^3^ Otitis has been separated from upper respiratory infections in the present paper because as described the temporary hearing loss caused by otitis does not allow some sounds to be heard. This abnormality in the sound signal quality leads to abnormalities with regard to distinguishing the phonetic coding. Other infections of the respiratory tract do not cause such disorders.

On the other hand, some researchers^4,5^ have shown that children who start to speak later and also present repeated episodes of otitis have a higher risk of presenting phonological disorder at 4:0 years (four years, zero months). This would occur because the temporary hearing loss that comes together with the otitis makes some sounds inaudible, since this loss is not the same for all frequencies.^6^ Despite the transitory nature of the hearing loss, it alters the sound perception quality because the auditory signal may be incomplete or inconsistent, thus inducing abnormalities in distinguishing the phonetic coding.^7^

The severity of phonological disorders may be manifested to differing degrees and thus give rise to variations in speech intelligibility.^1^ Another matter that has been the subject of much discussion among phonological disorder researchers is the extent to which its severity can be measured by indices. One of the purposes of such indices would be to measure the subject's improvement during the treatment and to indicate more precisely when the patient should be discharged.

There are many ways to quantify the severity of phonological disorders and to assist in differentiating the subtypes of phonological disorders in the literature.^8^ In 1982, two authors^1^ developed a procedure for evaluating the severity of phonological disorders called the percentage of consonants correct (PCC). This procedure investigates the number of correct consonants produced in a sample of continuous speech, in relation to the total number of consonants in this sample. These authors found that, for mild phonological disorders, the PCC was from 85 to 100%, while for mild-moderate disorders it was 65 to 85%, for moderate-severe disorders it was 50 to 65% and for severe disorders it was lower than 50%. In this method of counting, omissions, substitutions and common and uncommon distortions are considered incorrect.

Another way of measuring the severity of phonological disorders is the phonological density index (PDI).^9^ For this, the total number of phonological processes (defined as any systematic simplification of a sound class^10^) should be calculated and divided by the number of words analyzed in the sample. The PDI is unrelated to any specific type of speech sample and it can be used with any test or evaluation procedure. Although the PDI needs more refinement and more tests, it can be seen to have great clinical potential for measuring the severity and/or phonological intelligibility.

In 2002, the PCC and PDI were validated for Brazilian children.^11^ Other population studies using these severity indices have been carried out in the English language.^1,8,9^

In the Portuguese language, one study was performed, in which 22 subjects with phonological disorder were distributed according to the PCC severity classification. Only one occurrence of severe degree was observed; the majority of the occurrences were of mild-moderate degree (8 subjects), followed by moderate-severe degree (7 subjects) and mild degree (6 subjects). A negative correlation was observed between the two indices, thus showing that the PCC and the PDI were inversely proportional.^12^ Some other studies have also used the PCC in the Portuguese language.^11,13,14^

## OBJECTIVE

The aim of the present study was to investigate the variation in the PCC and PDI according to factors correlating with phonology disorders.

The hypothesis was that children with otitis and upper respiratory histories who began speaking later, were older at the time of the assessment period and were not comprehended would present worse severity indices.

## METHOD

This study was approved by the Ethics Committee for Research Project Analysis (CAPPesq) of the Clinical Board of Hospital das Clínicas, Universidade de São Paulo (protocol 286/99). The legal guardians of all the participating subjects signed an informed consent statement. This study was observational, analytical and transversal.

Fifty children with phonological disorders, aged between 4 and 11 years old, took part in this study. All the subjects were being attended by the Speech Language and Phonology Laboratory of the Department of Communication Disorder Sciences, Fa­culdade de Medicina da Universidade de São Paulo (FMUSP).

Regarding age, there were large numbers of subjects between 4:1 and 7:6 years old. The gender proportions were that 70% of the subjects were males (35) and 30% females (15), i.e. 2.33:1 (boys to girls). Moreover, most of the male subjects were between 4:7 and 5:0 years old, while the females were between 5:7 and 6:0 years old.

Firstly, the subject's parents were interviewed and an anamnesis was obtained, with some relevant data on the child's development. This included information such as whether the child was understood at the beginning of speech development and at the time when the disorder was diagnosed, history of respiratory diseases, history of otitis and age at which speech development began, among other data.

An audiological evaluation was carried out on all the children at the Audiology Service of the Speech and Hearing Department. The equipment used consisted of acoustic cabins, a Grason-Standler middle ear analyzer (model GSI-33), a Maico Hearing Instruments audiometer (model MA-21) and a Grason-Standler audiometer (model GSI-16).

All the subjects underwent speech assessment, including all the tests that form part of the Child Language Test (ABFW),^15^ an orofacial structure assessment and phonological awareness tests.

The phonology tests of imitation and naming were part of the Child Language Test (ABFW).^15^ These tests were recorded on a CD recorder with a Quicksound microphone connected. The recordings were made in an acoustically treated environment. The assessments were also recorded on digital video in order to enable better data analysis.

The PCC and PDI were calculated from the imitation and naming tests. These severity indices were calculated for each child.

For the PCC calculation, omissions, substitutions and common and uncommon distortions were considered to be errors for each subject. In the proposed classification,^1^ PCC scores over 85% were taken to be mild, scores between 65% and 85% were taken to be mild-moderate, scores between 50% and 65% were taken to be moderate-severe and scores less than 50% were taken to be severe.

The other severity measurement method was the phonological density index (PDI). This was calculated as the mean for the phonological processes of each word. The PDI took into account the fact that one incorrect sound might result in the application of one or more processes.^9^

Following data analysis, the group was divided into children with and without otitis and upper respiratory disorder histories; audiometric failure; whether they were or were not intelligible at the time of the assessment; age of onset of speech; and age at the time of the assessment.

The PCC and PDI were calculated and compared with the anamnesis data. The data were analyzed by means of calculating the PCC and PDI from the naming and imitation^14^ tasks, and these were compared with the anamnesis and audiological data that had been collected when the disorder was diagnosed.

In order to compare the children's performance in the tests, Student's t test for comparing means was used, with a significance level of 0.05. For associations of variables, Spearman's correlation was used.

## RESULTS

### Association between age and performance in the tests, using PCC and PDI

In relation to the children's ages (in months) at the time of the phonological assessment, for the PCC, Spearman's correlation was 0.468 in the imitation task (p = 0.001) and 0.431 in the naming task (p = 0.002); for the PDI, the correlation was 0.459 in the imitation task (p = 0.001) and 0.431 in the naming task (p = 0.002). Therefore, there was evidence for an association between the index values in the two tasks and the age at the time of the assessment, such that the higher the age was, the higher the PCC and the lower the PDI were.

In relation to the age at which the child began to speak, for the PCC, Spearman's correlation was 0.295 in the imitation task (p = 0.064) and 0.312 in the naming task (p = 0.050). For the PDI, the correlation was 0.292 in the imitation task (p = 0.067) and 0.323 in the naming task (p = 0.042). This showed that there was a correlation between the indices and the age at which the child started to speak. Therefore, the later that speech development began, the lower the PCC and the higher the PDI were. It is important to stress that the p values were very close to 0.05, thus indicating that in a larger sample, the p values could be < 0.05. If a significance level of 0.10 were taken, these values would be significant.

### Difference between PCC and PDI among subjects with and without upper respiratory tract infection and otitis history in the tests

**Figure 1:**
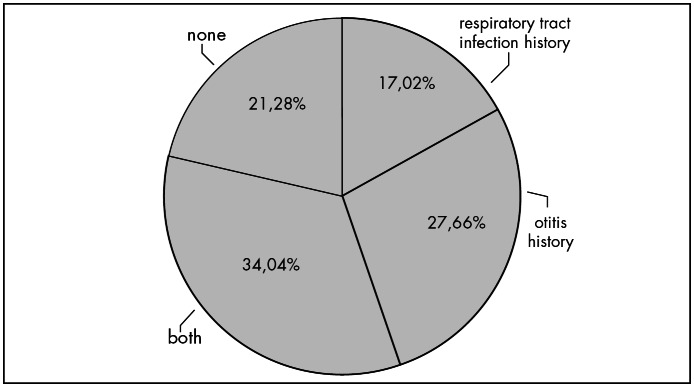
Subject distribution according to otitis and history of respiratory tract abnormalities.

Figure 1 shows the subject distribution regarding histories of otitis and respiratory abnormalities.

Tables 1 and 2 show, respectively, the mean PCC and PDI for subjects with and without upper respiratory tract infections and otitis history. Slight variation in the indices analyzed was seen.

**Table 1:** Percentage of consonants correct and phonological density index descriptions according to the presence/absence of history of respiratory abnormalities

Was the subject understood?		n	Mean	Standard deviation	Maximum	Minimum
Yes	PCC imitation	35	84	11.6	97.2	45.8
	PCC naming	35	83.5	12.3	97.7	40.2
	PDI imitation	35	0.5	0.4	2	0.1
	PDI naming	35	0.5	0.4	1.9	0.1
No	PCC imitation	10	79.1	6.6	92.5	69.2
	PCC naming	10	78.7	10.8	97.8	65.6
	PDI imitation	10	0.6	0.2	1	0.1
	PDI naming	10	0.6	0.3	1	0.1
Total	PCC imitation	45	82.9	10.8	97.2	45.8
	PCC naming	45	82.4	12.1	97.8	40.2
	PDI imitation	45	0.5	0.4	2	0.1
	PDI naming	45	0.5	0.4	1.9	0.1

PDI = phonological density index^1^; PCC = percentage of consonants correct.^18^

**Table 2:** Percentage of consonants correct and phonological density index descriptions according to the presence/absence of otitis history

Otitis		n	Mean	Standard deviation	Maximum	Minimum
Yes	PCC imitation	30	82.2	11.3	97.2	45.8
	PCC naming	30	82.2	12.74	97.7	40.2
	PDI imitation	30	0.5	0.37	2	0.1
	PDI naming	30	0.5	0.4	1.9	0.1
No	PCC imitation	18	79.6	12.29	96.3	56.1
	PCC naming	18	78.3	12.65	97.8	56.3
	PDI imitation	18	0.58	0.38	1.6	0.1
	PDI naming	18	0.58	0.36	1.2	0.1
Total	PCC imitation	48	81.2	11.61	97.2	45.8
	PCC naming	48	80.7	12.71	97.8	40.2
	PDI imitation	48	0.52	0.37	2	0.1
	PDI naming	48	0.53	0.38	1.9	0.1

PDI = phonological density index^1^; PCC = percentage of consonants correct.^18^

The mean PCC and PDI obtained in the imitation and naming tasks were calculated and compared with the presence and absence of respiratory abnormalities. Firstly, Levene's variance comparison test was used to choose which would be the most appropriate version of Student's t test. Thus, the values found in Levene's comparison were: for the PCC in the imitation task, p = 0.136, and in the naming, p = 0.530. Then, the equal variance Student's t test was applied with the following results: p = 0.016 and p = 0.005 respectively. For the PDI, the values obtained in Levene's test were p = 0.032 in the imitation task and p = 0.018 in the naming task; the unequal variance Student's t test was also applied with the following results: p = 0.014 and p = 0.008, respectively. Thus, Student's t test showed that there were differences in the mean performance of the groups with and without upper respiratory tract infections, in relation to both the PDI and the PCC, in both tasks.

The same kind of analysis was performed for the history of otitis media. For the PCC, Levene's test gave p = 0.493 for imitation and p = 0.594 for naming, and for the PDI, this test gave p = 0.600 for imitation and p = 0.734 for naming. Thus, the equal variance Student's t test was applied and the values found were: for the PCC, p = 0.466 in the imitation task and p = 0.318 in the naming task, and for the PDI, p = 0.460 in the imitation task and p = 0.507 in the naming task. Taking a significance level of 0.05, there was no difference between the means, in either group.

### Differences between mean PCC and PDI for subjects with and without audiological abnormalities

Table 3 indicates the mean PCC and PDI for subjects with audiological abnormalities.

**Table 3:** Percentage of consonants correct and phonological density index descriptions according to the audiological evaluation result

Audiological evaluation abnormality		n	Mean	Standard deviation	Maximum	Minimum
Yes	PCC imitation	3	82.9	2.2	84.1	80.4
	PCC naming	3	82.4	4.8	87.8	78.4
	PDI imitation	3	0.5	7.6	0.5	0.4
	PDI naming	3	0.5	0.1	0.5	0.3
No	PCC imitation	28	84.2	10.8	97.2	45.8
	PCC naming	28	83	12.6	97.8	40.2
	PDI imitation	28	0.4	0.36	2	0.1
	PDI naming	28	47	0.39	1.9	0.1
Total	PCC imitation	31	84.1	10.3	97.2	45.8
	PCC naming	31	83	12	97.8	40.2
	PDI imitation	31	0.4	0.3	2	0.1
	PDI naming	31	0.5	0.4	1.9	0.1

PDI = phonological density index^1^; PCC = percentage of consonants correct.^18^

The mean PCC and PDI in the naming and imitation tasks were compared between the subjects with and without audiological abnormalities. For the PCC, Levene's test gave p = 0.122 for imitation and p = 0.200 for naming, and for the PDI, p = 0.256 for imitation and p = 0.209 for naming. Therefore the equal variance Student's t test was applied and the following values were found: for the PCC, p = 0.831 in the imitation task and p = 0.936 in the naming task, and for the PDI, p = 0.900 in the imitation task and p = 0.967 in the naming task. Taking a significance level of 0.05, there was no evidence of differences in the naming and imitation task performance between the groups with and without audiological alteration.

### Comparison between the mean PCC and PDI for subjects with and without problems of being understood when they started to speak

Table 4 shows the mean PCC and the PDI according to the comprehension of the subjects’ speech when they started to speak.

**Table 4:** Percentage of consonants correct and phonological density index descriptions according to the comprehension of the subject's speech at the beginning of speech

Was the subject understood?		n	Mean	Standard deviation	Maximum	Minimum
Yes	PCC imitation	22	81.9	12.5	96.3	45.8
	PCC naming	22	80.4	13.3	95.6	40.2
	PDI imitation	22	0.5	0.5	2	0.1
	PDI naming	22	0.5	0.4	1.9	0.1
No	PCC imitation	14	79.9	9.3	92.5	57.9
	PCC naming	14	81.1	12.2	97.8	56.6
	PDI imitation	14	0.5	0.3	1.2	0.1
	PDI naming	14	0.5	0.4	1.5	0.1
Total	PCC imitation	36	81.1	11.3	96.3	45.8
	PCC naming	36	80.7	12.7	97.8	40.2
	PDI imitation	36	0.5	0.4	2	0.1
	PDI naming	36	0.5	0.4	1.9	0.1

PDI = phonological density index^1^; PCC = percentage of consonants correct.^18^

Levene's test gave the following values: for the PCC, p = 0.408 in the imitation task and p = 0.895 in the naming task, and for the PDI, p = 0.501 in the imitation task and p = 0.794 in the naming task. The equal variance Student's t test did not show any evidence of differences between the performances of the two groups: for the PCC, p = 0.621 in the imitation task and p = 0.871 in the naming task, and for the PDI, p = 0.920 in the imitation task and p = 0.959 in the naming task.

### Difference between the mean PCC and PDI for subjects with and without problems of being understood at the time of the assessment

Table 5 shows the mean PCC and PDI for the subjects according to the comprehension of their speech at the time of the speech-language assessment and when they started to speak. Slight variation in the indices analyzed can be seen.

**Table 5:** Percentage of consonants correct and phonological density index descriptions according to the comprehension of the subject's speech during the speech-language evaluation period

Was the subject understood?		n	Mean	Standard deviation	Maximum	Minimum
Yes	PCC imitation	35	84	11.6	97.2	45.8
	PCC naming	35	83.5	12.3	97.7	40.2
	PDI imitation	35	0.5	0.4	2	0.1
	PDI naming	35	0.5	0.4	1.9	0.1
No	PCC imitation	10	79.1	6.6	92.5	69.2
	PCC naming	10	78.7	10.8	97.8	65.6
	PDI imitation	10	0.6	0.2	1	0.1
	PDI naming	10	0.6	0.3	1	0.1
Total	PCC imitation	45	82.9	10.8	97.2	45.8
	PCC naming	45	82.4	12.1	97.8	40.2
	PDI imitation	45	0.5	0.4	2	0.1
	PDI naming	45	0.5	0.4	1.9	0.1

PDI = phonological density index^1^; PCC = percentage of consonants correct.^18^

Levene's test for the PCC gave p = 0.232 for imitation and p = 0.947 for naming, and for the PDI, p = 0.379 for imitation and p = 0.883 for naming. Therefore, the equal variance Student's t test was applied, which did not show any evidence of differences between the subjects whose speech was and was not comprehended at the time of the assessment: for the PCC, p = 0.212 in the imitation task and p = 0.276 in the naming task, and for the PDI, p = 0.351 in the imitation task and p = 0.264 in the naming task.

## DISCUSSION

Since phonological disorders may be identified and treated before children reach four years old, this places a great responsibility on the health professionals that deal with this population. It must also be stressed that, according to the DSM-IV (Diagnostic and Statistical Manual of Mental Disorders),^16^ there is a great possibility that children with phonological disorders will develop problems in learning to read and write. Therefore, early detection of such disorders would improve the chances of better academic performance.

The results found from this study indicate that certain factors that correlate wi­th phonological disorders are associated wi­th increased severity of such disorders. A relationship could be seen between the PCC and the PDI obtained from the naming and imitation tasks of the Child Language Test (ABFW)^15^ and the following factors that correlate with phonological disorders: age when speech development began, history of otitis and upper respiratory tract infections, audiometry data and understanding of the child's speech at the beginning of speech development and at the time of the evaluation.

The subjects’ age at the time when the phonological disorder was diagnosed had a significant correlation with the PCC and PDI, such that the greater the age was, the higher the PCC and the lower the PDI were. This indicates that younger children present a more severe picture and suggests that a certain degree of speech normalization takes place among children who are thus diagnosed later on.^17,18^ However, it also indicates that, without speech-language intervention, children with phonological disorders do not attain adult speech patterns.

A significant correlation was found between the PCC and PDI and the age at which the child started to speak, thus demonstrating that the later that the child started to speak, the lower the PCC and the higher the PDI were. This same finding was observed in another study,^19^ on the PCC-R index, in which the results showed that children classified as presenting delayed speech development had lower performances than did children with typical development.

The presence of otitis media and/or upper respiratory tract infections was observed in 79% of the population. According to the data in the literature, this is a common finding and is probably indicative of a phonological disorder subtype.^20,21^

With regard to upper respiratory tract abnormalities, a statistically significant difference was found between the groups with and without such histories, for both the PCC and the PDI. The descriptive analysis showed that there was better performance, as shown by the PCC and PDI, in the group without upper respiratory tract abnormalities.

No significant differences were found in relation to previous otitis history between the mean PCC and PDI for the groups with and without such histories. Some authors^22-26^ did not find any direct relationship between such abnormalities and phonological performance or the severity of the phonological disorder. In the present study, the descriptive analysis showed that children with a previous history of otitis presented lower PCC and PDI, thus indicating the presence of more phonological disorders in those children.

A study using the PCC-R^19^ severity index revealed that English-speaking children classified as presenting delayed speech development were on average 15% behind children with typical development.

In a previous study,^27^ the PDI was used to investigate the severity of phonological disorders among children with histories of otitis media or upper respiratory tract infections. The analysis indicated that the mean PCC in the phonology tasks of the Child Language Test (ABFW)^15^ did not present significant differences between the groups with and without histories of otitis and/or upper respiratory tract infections.

No statistically significant differences in PCC and PDI values were found between subjects with and without abnormalities in the audiological evaluation at the time when their phonological disorder was diagnosed. This result was expected, since the otitis episodes occur most frequently during the first two years of life, and the mean age of the subjects in the present study was six years and five months. Therefore, the time when the greater disorders are found in audiological evaluations had already passed.

There was no statistically significant difference with regard to comprehension or lack of comprehension of the child's speech by his family at the time the child started to speak or at the time of the evaluation, i.e. no difference in the PCC and PDI between children who were or were not understood. Despite this, the descriptive analysis indicated that children who were not understood at the time of their diagnosis had a lower PCC and a higher PDI than did those who were understood. This shows that the segmental features that are added to the supra-segmental features of prosody and voice contribute towards a certain degree of speech unintelligibility perceived by the listener, as stated by some authors.^1^

The present study corroborates the finding from other studies^3,22,28-32^ that children with several otitis and upper respiratory tract infection episodes show phonological disorders characterized by different degrees of speech unintelligibility. Early intervention in cases in which phonological disorders are perceived speeds the normalization of the phonological system and decreases the harm caused by such disorders.

The results from the present study corroborate the opinion expressed in other studies^22,28^ that further studies on the causal factors associated with phonological disorders are needed.

## CONCLUSION

Certain factors studied, which correlate with phonological disorders, showed associations with the severity indices. The greater the age at the time of the assessment was, the higher the PCC and the lower the PDI were. On the other hand, the later that speech development began, the lower the PCC and the higher the PDI were. Also, the severity indices differed between the groups of subjects with and without histories of upper respiratory tract infections. The other factors studied did not show any associations with the severity indices.

These pointers constitute a warning sign for health professionals who deal with preschool children. Children with delayed speech development or a history of chronic upper respiratory infections must be referred for speech-language evaluation, in order to investigate whether phonological disorders are present. Early diagnosis enables brief and efficient intervention, thereby avoiding other disabilities of a social and scholastic nature.
